# Applications of propolis-based materials in wound healing

**DOI:** 10.1007/s00403-023-02789-x

**Published:** 2023-12-27

**Authors:** Mohamed El-Sakhawy, Ahmed Salama, Hebat-Allah S. Tohamy

**Affiliations:** Cellulose and Paper Department, National Research Centre, 33 El Bohouth St., Dokki, P.O. 12622, Giza, Egypt

**Keywords:** Propolis, Composite, Wound healing

## Abstract

Due to its excellent antiseptic efficacy and antimicrobial properties, propolis has shown attractive advantages in wound dressings. However, an inclusive review of the propolis-based materials as a wound dressing is still lacking. The current short review summarizes the skin wound healing process, relates evaluation parameters, and then reviews the refined propolis-based materials dressings such as antimicrobial property, adhesion and hemostasis, anti-inflammatory and substance delivery. The approaches implemented to achieve these functions are classified and discussed. Furthermore, applications of propolis wound dressing for treating different types of wounds such as heal wounds, burns, and ulcers are presented. The future directions of propolis-based wound dressings for wound healing are further proposed. This review showed that propolis-based materials might be a promising new dressing for wound occlusion and tissue repairing.

## Introduction

Propolis is a well-known natural sticky, resinous material collected by bees from buds and the bark of different tree species and mixed with bee enzymes, pollen, and wax. Propolis is also known as bee glue, as bees use it to smooth out internal walls, seal holes, and close gaps in their honeycombs. A sterile environment protects the colony from diseases because of its antiseptic efficacy and antimicrobial properties [1]. Bees use propolis also to embalm carcasses of invaders such as other insects who are died inside the hive, avoiding their decomposition [2]. Propolis has become the subject of several studies carried out worldwide in the last decades. Many reviews have discussed the propolis’s chemical composition and biological properties. Propolis can be considered as a complex mixture of chemicals. The composition of propolis varies greatly depending on the time of collection and the plant’s raw material constituents. Usually, propolis comprises 50% resin (flavonoids and phenolic acids), 30% waxes, 10% essential oils, 5% pollen, and 5% other compounds. Ethanol has been the most used solvent to obtain low wax propolis extracts rich in biologically active compounds [3].

Propolis has been widely used in traditional and complementary medicine to treat wounds and ulcers because of its antiseptic and local anesthetic properties. It has been used for a long time in medicine owing to its bactericidal, antiviral, antifungal, anti-inflammatory, antitumor, immunomodulatory, and antioxidant activities [4]. The ancient Egyptians, Greeks, and Romans were the first to use propolis for wound healing and as a disinfection substance [5]. Moreover, ancient Egyptians used propolis for mummification as a preservative agent; propolis was one of the most important constituents [6]. Recently, propolis has been proven safe and non-toxic for human use. It is considered a promising natural source for new pharmaceutical products.

## Wound healing process

The natural wound healing process in the body involves multiple biochemical and cellular reactions, which occur in four successive main stages: hemostasis (coagulation), then inflammation, followed by proliferation of new skin tissue, and finally remodeling of mature tissue [7].

### Hemostasis

Hemostasis is the first natural response that takes place immediately after skin injury. The blood platelets accumulate at this stage in the place of injury and release a chemical warning sign. As a result of these signs, the fibrin, a blood protein, forms a glue web that binds the platelets to form blood clots (thrombi). The formed blood clots seal the wound and diminish the bleeding. Propolis could control the early stages of wound healing, as in the hemostasis stage [8].

### Inflammation

The inflammation stage starts at once following the blood clot formation. Inflammatory cells such as neutrophils excrete huge amounts of reactive oxygen species (ROS), whilst immune cells such as macrophages excrete huge amounts of pro-inflammatory cytokines. Neutrophils and then macrophages arrive at the wound site to stall the chronic wound healing in the inflammation stage. The produced ROS act as a defense system against pathogens in the wound area. Nevertheless, any excess ROS leads to many disorders without a balance between production and deactivation. Free radicals can damage the surrounding normal tissue (as in diabetes) and oxidize cell lipids, proteins, and nucleic acids. The protective effects of propolis polyphenols in wound healing are different; they inhibit the appearance of ROS, chelate metals ions involved in the ROS formation, and scavenge ROS, thus reducing the excessive accumulation of ROS and hindering the peroxidation of lipids and tissue [9].

### Proliferation

The proliferative stage occurs after the injury by 2 or 3 days. The proliferative stage itself included four stages: epithelization, angiogenesis, granulation, and collagen deposition. The epithelization stage involves the epithelial cells’ movement from either migration side to get together. It takes place by inspiration of fibroblasts in the dermis layer beside keratinocytes in the epidermis. The angiogenesis stage involves the formation of new blood vessels from pre-existing vessels to supply oxygen and nutrition required to repair the injured tissue. Then, in the granulation stage, proliferative fibroblasts start to form matrix proteins and collagen to construct the extracellular matrix that acts as a new connective tissue and microscopic blood vessels to form primary dermal granulation tissue on the wound surfaces. Finally, in the collagen deposition stage, mainly in the third week after injury, the proliferation of the particular fibroblasts, called myofibroblasts, causes contraction of the wound size due to the increase in collagen synthesis compared with the proliferative process. Propolis contains active compounds promoting dermal cell proliferation, activation, and growth [10].

### Remodeling

The final stage of wound healing is the remodeling and maturation stage. The new epithelium and scar tissue are formed due to the replacement of type III collagen with type I collagen. The collagen deposition in this stage is more organized; the collagen fibers are thicker and aligned with the original collagen fibers of the skin. This stage may last up to a year or longer to complete.

Propolis phenol compounds, including flavonoids and phenolic acids, can accelerate proliferation, remodeling, and maturation. It was reported to stimulate the repair of granulation cells in burnt dermal tissue. Also, propolis encourages collagen types I and III expression and degradation in the wound matrix, supporting re-epithelization. Several studies confirmed propolis as a promising material to aid wound healing by activating all involved stages [11].

## Wound healing properties of propolis component

Due to their renewable nature, biocompatibility and biodegradability, there has been an increasing concern about using biomaterials in healthcare products in the last decades. Applying wound dressing for wound healing has increased attention [12]. Intriguingly, propolis exhibits antimicrobial, antioxidant, and anti-inflammatory effects, making it the right product for wound healing. Propolis has extra advantages over ordinary antimicrobial agents, like silver, such as high biocompatibility, natural origin, and plasticizing properties that improve the film’s flexibility and processability.

Propolis contains enormous active components that promote healing and is commonly used in folk medicine. The positive biological activity of propolis on tissue regeneration and wound repair results from its polyphenolic flavonoids’ anti-oxidative, anti-inflammatory, antimicrobial,

and immunomodulatory properties. Caffeic acid in propolis extracts also proved to have potent antioxidant and anti-inflammatory activities, leading to wound healing in mice. Moreover, propolis shows an antibacterial effect against clinical wound isolates and is synergistic with some antibiotics [13, 14]. In addition, propolis accelerates the wound healing process in diabetics people, immune-suppressed patients, and patients of advanced age [15]. Propolis has an excellent effect on burns healing management as an external treatment. It enhances skin cell proliferation, activation, and growth ability. The healing effect of propolis on connective tissue fibroblasts was verified. Propolis could motivate a constructive biochemical environment appropriate to re-epithelization. Propolis can inhibit the formation or disrupt the oral or dental biofilms [16, 17]. Propolis has various action approaches, giving it an exceptional opportunity for therapeutic achievement than common drugs.

## Propolis role in the wound healing process

Wounds disrupt the skin continuity, which may happen by burns, trauma, incision, or a medical reason. Microorganisms such as bacteria and fungi can colonize the wound site, causing infection, delaying the healing, and increasing the local tissue damage. Propolis properties and biological activities such as antimicrobial, remodeling of the skin tissue, and skin cell proliferation enhancer were related to skin healing promotion. In particular, propolis was recommended in folk medicine for wound healing, skin regeneration, treatment of purulent diseases, relief of all kinds of pain, and local anesthesia. Propolis demonstrates excellent therapeutic efficacy in different wound types, such as diabetes, infection, surgical, burns, and gastric ulcers [18]. Wound healing proceeds through a finely tuned pattern of successive stages, such as hemostasis (coagulation), inflammation, cell proliferation, and tissue remodeling.

Propolis can reduce the inflammatory response to achieve a better healing process. Propolis treatment significantly increases the extracellular matrix components during the early wound healing stage; then, the extracellular matrix molecule reduction was observed. Propolis stimulates the expression of transforming growth factor-β (highly pleiotropic cytokine) that contributes to the initial stages of wound repair, such as hemostasis and inflammation.

The harmful effect of free radicals in skin and tissues could be suppressed by propolis via the quench of free radicals in the skin. This effect of propolis on free radicals in the skin is beneficial for burn wound therapy. Propolis inhibits ROS formation, which inhibits the eicosanoid synthesis, retards NF-κB activation, reduces expression of different inflammatory cytokines, and inhibits the oxidative damage to carbohydrates, proteins, lipids, and DNA/RNA.

A study on diabetic rodents showed the significant effects of propolis in the acceleration of wound healing rate. Propolis supports the re-epithelialization process and decreases the inflammation by normalizing the physiological count of neutrophil and macrophage flow into the wounded area, which prevents the persistent inflammation usually observed in diabetes. Propolis in topical cream compositions showed similar results [19].

Propolis is considered a promising material for burn healing via promoting skin cell proliferation, activation, and growth ability, with no toxicity and rare allergic effect. Propolis activity in burns healing essentially occurs via organizing the immune response through the inflammatory stag [20]. The skin permeation by a local propolis ointment formation on rodents’ dermal wounds relies on the healing phase. It mostly stimulates the proliferation stage of wound healing by motivating the formation of keratinocytes. This ointment reduced wound area more efficiently than typical preparation [4]. The local application of propolis reduces the number of mast cells and neutrophils, accelerating the revival process and wound healing [21].

Propolis cream enhances dermal tissue healing in patients and decreases wound inflammation more efficiently than silver sulfadiazine local treatment. Propolis encouraged epithelial cellular repair of mouth injury [22]. Also, corneal epithelial wound healing in mice was repaired by applying 1% water extract of propolis, and the epithelial defect area was smaller in contrast to the control group. Thus, propolis is considered a potent wound healing accelerator due to its broad-spectrum activity during all wound healing stages.

Collagen synthesis is a main and vital stage required for wound contraction and associated with the healing process. Propolis accelerates the expression of collagen type I during the early stage of wound healing and enables wound closure [11]. In a type I diabetic mouse model, local application of propolis shows accelerated wounds healing and closure of diabetes. Propolis promotes wound closure by increasing expression and deposition of collagen type I, reducing matrix and decreasing inflammation [11]. Propolis is also beneficial to accelerating the burned tissue healing; it stimulates remodeling of the wound extracellular matrix due to its flavonoid constituent, which prevents necrosis of cells and reduces lipid peroxidation [23]. This process involves the migration and proliferation of keratinocytes and epidermal cells, fibroblast adhesion, and extracellular matrix constriction [24].

Propolis is useful for abolishing the infection during wound treatment due to its immune-stimulating effect [25]. Propolis alters the bacteria cell membrane permeability, inhibits the synthesis of some proteins, and inhibits cell division, so it is an effective antibacterial [26].

Propolis reduces scar formation after wound healing, increases wound contraction, reduces the healing time, and stimulates tissue repair. Biofilm formation is an important factor that can deteriorate wound repair during one or more stages of wound healing. Propolis is considered a promising biomaterial in treating wounds biofilm. Synergistic prospects between propolis and antibiotics have been confirmed [20].

## Propolis composites for wound healing

Wound dressings are biomaterials employed to cover the wounds (physical barrier), which can absorb exudates, maintain the moisture balance, permit gas exchange, and inhibit the wounds from being infected. An ideal wound dressing should exhibit specific properties, including flexibility, durability, wound hydration/dehydration, and appropriate mechanical properties [27]. Moreover, ideal wound dressings should present properties (physicochemical and biological) to the treated area and degradation rate on a time scale compared to wound healing [28]. Wound dressings fabricated from natural polymers (e.g. proteins and polysaccharides) have grown due to their properties compared to other dressings [29]. This review aims to evaluate the biodegradable natural materials/propolis in wound care. Propolis was used in various formulations such as ethanol extract of propolis, water extract, and powder of native propolis [30]. Table 1 summarizes some recent studies concerning propolis composite utilized in different forms and formulations and its effect and benefit as wound healing material.

**Table 1:** Propolis composites and its effect and application for wound healing

Propolis composite	Propolis effect	Applications	References
Dressings with propolis in 2% ointment	Inhibit nuclear transcription factor kappa-b and reducing the levels of inflammation, pain, and microbial load. Prevent adhesion to the tissues in formation of the wound and effective against biofilm	Favors healing with a humid environment, used with venous ulcers, pressure ulcers and diabetic foot	[31]
Green propolis-based ointment. petrolatum (81.6%) and propolis 11.5%	Improve hematomas in the flaps and periflaps of skin tears. Prevent the colonization and proliferation of bacteria at the injury site, recovering the injured tissue and resulting in viable and quality tissue	Treatment of skin tears in an elderly hospitalized population	[32]
Propolis nanostructured lipid carriers (4 ml) loaded on Carbopol gel (0.5 g)	Increased the phenolic and flavonoid contents. Antioxidant activities increased by 25-fold. Twofold higher antimicrobial effect, higher skin regenerative potency, fast wound closure and minimal scar formation	Accelerate the healing of the wound, protection from microbial contamination	[33]
Collagen hydrolysates with honey-propolis wax, structurally modified as a sponge matrix	Regulated wound biochemical markers and upregulated the expression of growth factors, potential capacity to perform wound re-epithelialization and the loading of ground tissue. A significant reduction in inflammation and increased the loading of collagen	Enhanced and faster wound healing process	[34]
Ethanolic propolis extract and honey	Improve cell migration, cell viability, and proliferation of HDF cells	Improve wound healing	[35]
Propolis (0.4%) and tea tree oil nanoemulsion loaded with clindamycin hydrochloride	Enhances re-epithelialization, collagen production and has potent anti-inflammatory properties	Efficient local therapy for heal wound effectively	[36]
PVA/Chitosan- membrane scaffolds loaded with propolis (0.25, 0.50, % v/v)	Promoted cell proliferation and adhesion; and have no genotoxic potential	Scaffolds for wound healing applications	[37]
Flexible sodium hyaluronate films with encapsulated propolis (8–16%)	An increase in hydrophobicity and improvement in mechanical and thermal properties of the obtained films	Effective dressings to be applied on infected wounds and dermatology	[38]
Propolis nanoparticles (PNPs)	Free radical scavenging potential, powerful antioxidant activity and anti-inflammatory effects, enhanced collagen production and angiogenesis, thus actively promoting skin wound healing	Promising candidates for clinical application in skin wound healing	[39]

### Textiles and nanofibers

Natural materials are sensible approaches to bioactive fibers with functional properties for biomedical [40] and environmental applications [41, 42]. Propolis, as an emulsion, has been successfully used on a textile substrate. For example, propolis with beeswax and chitosan emulsion on cotton fabric was used for wound healing in rats. Also, pure cotton knitted fabric was treated with antimicrobial skin care emulsion in a mixture with propolis extract [43]. Propolis was used effectively in practical therapeutics to accelerate cell proliferation and wound healing. Special dressings containing propolis were used for burning wound treatment to enhance epithelization and reduce infection risk.

Polypropylene yarns soaked with propolis were fabricated, and the propolis components released from yarns and their potential cytotoxicity on cell formation were investigated. Results show that natural propolis extract additive could be successfully used to develop healing textile appropriate for biomedical applications [44].

**Fig. 1:**
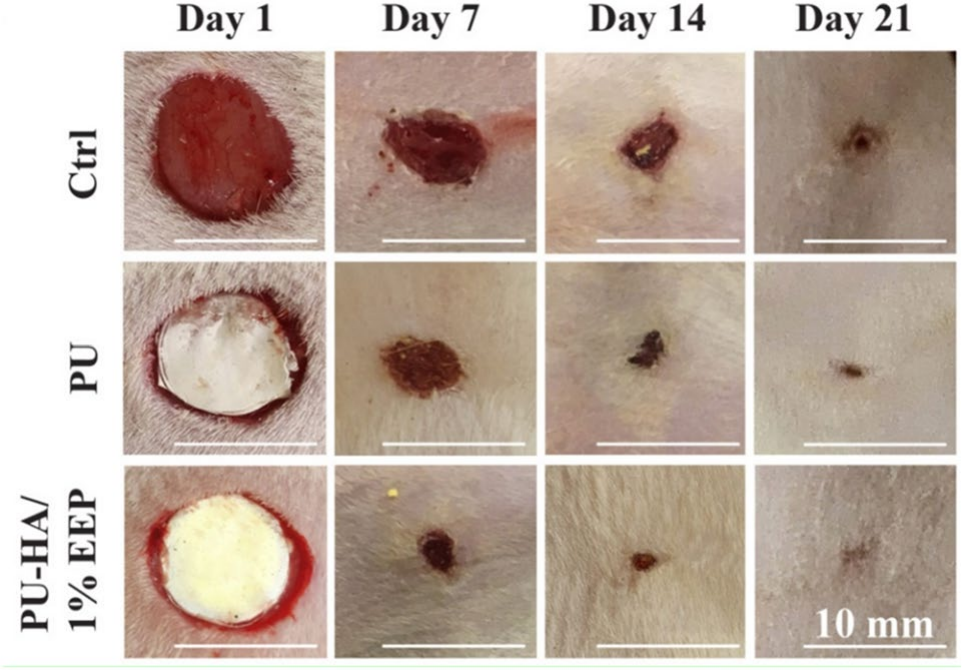
Effect of propolis on wound healing process

Electrospinning of propolis-enriched nanofibers for biomedical applications has a growing concern in achieving electrospun wound dressings and antibacterial scaffolds [45]. Wound dressing with enhanced efficacy was fabricated by electrospinning technique from polyurethane or polyurethane-hyaluronic acid nanofibers loaded with propolis ethanolic extract. This scaffold displays promising properties for wound healing, considerable biocompatibility, and antibacterial activities for wound dressing and advanced biomedical applications [46, 47]. The addition of 1% ethanolic extract of propolis (EEP) to polyurethane-hyaluronic acid (PU-HA) nanofibrous wound dressing obviously accelerates the wound healing process, as shown in Fig. 1 [34]. The hydrophilicity nature of the polyurethane was improved by adding propolis and showed excellent cell compatibility desired for wound dressing applications. Moreover, electrospinning of biocompatible polyurethane nanofibers loaded with propolis was productively prepared as a scaffold in tissue engineering to produce the proper environment for cell migration [48].

Electrospun polylactic acid fibers integrated with propolis were investigated to be applied as a wound dressing. These fibers show antibacterial activities from propolis and suitable air permeability to provide the skin with moisture, so it is more effective for healing wounds than traditional wound dressings [49]. Electrospun mats shaped from cellulose acetate solution loaded with propolis extract were evaluated as wound healing mats with antibacterial properties. This bio-composite has a prospective vital application due to its sustained release of propolis from the nanofibers mat accompanied by effective inhibition of bacterial growth [48]. Polyvinylpyrrolidone electrospun nanofibers with dispersed propolis and Aloe vera were also studied. Increasing propolis concentration decreased the surface tension and viscosity, which was valuable for electrospinning. The amazing properties of propolis, such as accelerating the wound healing process, burn treatment, and tissue repair, recommend it as an exceptional candidate for tissue engineering [50].

Table 2 summarizes some recent studies concerning electrospun fibers and propolis composite and its effect and benefit as wound healing material.

**Table 2:** Propolis electrospun fibers composites and its effect and application for wound healing

Propolis composite	Propolis effect	Applications	References
Bilayer wound dressing consisting of poly-ε-caprolactone/chitosan electrospun fibrous mat as the sublayer and a polyurethane foam coated with EEP (50 g propolis in 250 ml ethanol) as the top layer	Promoted the mechanical stability and antibacterial properties of the fabricated wound dressing. Significantly and effectively accelerate cell compatibility and the healing process	Used as a wound dressing because of its biocompatibility, and wound healing properties	[51]
Cellulose acetate nanofiber, chitosan, and propolis	Excellent hydrophobicity, cytotoxicity and cell viability. After 21 days of treatment with the loaded nanofibers; repairing of epithelial cells, hair follicles and sebaceous glands in the skin of the burn wound were found in mice model	A smart bandage provided with control of drug release as a burn wound healing scaffold	[52]
pH-sensitive propolis (10–30% v/v)-loaded electrospun Eudragit^®^ L-100 (EU)/ HPMC nanofibers	Cytocompatibility, cell attachment, and the antibacterial properties improved. The prepared mats had no toxic effect on fibroblast cells, and also caused cell growth. It is hemocompatible and they showed no toxicity to erythrocytes	EU/HPMC/PRO nanofibers had good potential to be used as a wound dressing	[53]
Electrospun PVA nanofbre loaded with green propolis extract, chitosan and nystatin	Decreases the viscosity of the PVA blank solution, altered the fibres’ morphological characteristics, exhibiting a decrease in fbre diameter	A novel material for wound and burn dressings or for transdermal delivery systems	[54]
Electrospun silk fibroin and gelatin nanofibers with propolis extracts (0.5–3%)	Improved antibacterial properties, biocompatibility, and can support cell adhesion and growth for a long time. Provide good migrating-promoting capability, and healing-promoting ability	Trauma dressings promise for skin regeneration therapy	[55]
Electrospun films formed by PCL fibres, beaded fibres, and beads containing alcoholic propolis extract (15% v/v)	Acted as a lubricant agent, affecting the spun solutions’ viscosity and the thermal properties of the mats. Increased the mats’ hydrophilicity and promote a better environment to support cellular events during the wound healing process	Good potential in improved wound dressing applications	[56]
Propolis Ethanolic Extract (PEE) with poly-ε-caprolactone (PCL) were blended to prepare elastomeric electrospun mats	Good mechanical and antimicrobial properties, and accelerated wound dressing ability	wound dressing and Guided Tissue Regeneration (GTR) membranes	[57]
Electrospun polyurethane-based nanofibers inclusive Calendula officinalis and Propolis ethanolic extracts	The favorable antibacterial effect of the nanofibers against MRSA bacteria was confirmed. Boost wound healing process with notable antibacterial, antioxidant, and cell proliferation properties	Antibacterial wound dressing to patients suffering from infectious wounds	[58]

### Hydrogels

Hydrogels containing anti-microbials are a favored class of materials for wound drug delivery and healing applications [59–61]. Hydrogels provide a humid environment and help autolytic debridement. Hydrogels containing suitable antimicrobial properties by incorporating bactericidal substances are required for the new generation of wound dressing materials. Flavonoids and phenolic compounds can be incorporated into the hydrogel matrix to improve antioxidant, anti-inflammatory, anticancer, and antiviral activities [62].

A mixture of κ-carrageenan and β-cyclodextrin as a biodegradable polymeric hydrogel was prepared to encapsulate propolis extract. Glyoxal was used as a crosslinking agent to increase the insolubility of the propolis-loaded hydrogel for cotton fabric treatments. Antimicrobial activities, the release profile of propolis, biodegradability, and extreme properties of polysaccharide blend gel are recommended for wound dressing application [63].

The covered wounds in a rat model with a polyacrylic acid hydrogel containing propolis showed rapid wound healing. The hydrogel shows a good contraction and closure effect and allows propolis for diverse topical applications [64].

**Fig. 2:**
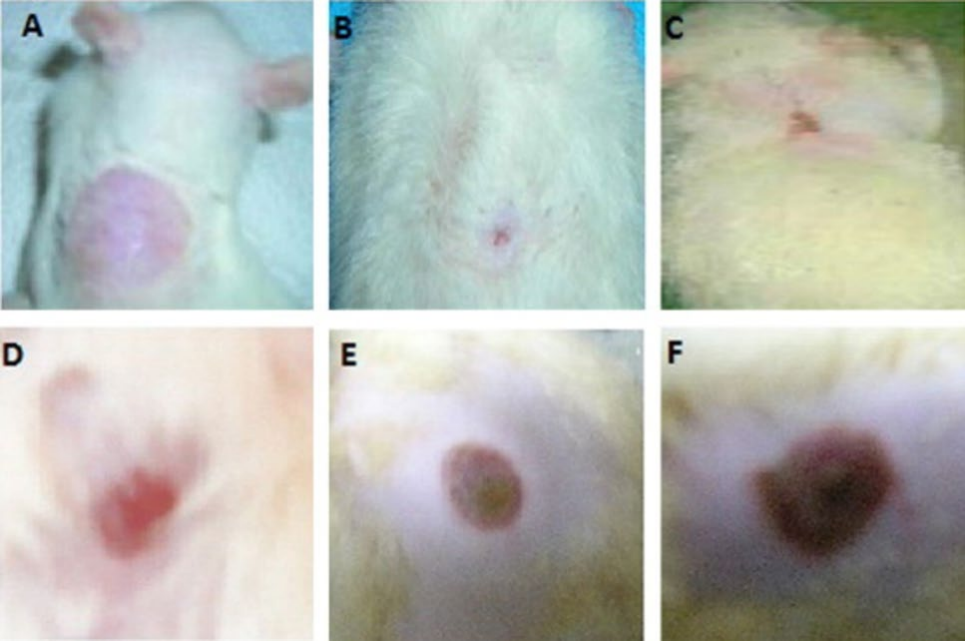
Photograph showing burn wound healing in rats showing **A** wound on day 0 and wound closure on 18th day for **B** silver sulphadiazine as a control, **C** Propolis loaded AgNP gel, **D** physical mixture of propolis and AgNP gel, **E** AgNP gel and **F** negative control

A hydrophilic lipid (Gelucire) was used to load the propolis lipophilic components onto the in situ silver nanoparticles’ surface. When formulated into a gel, these loaded nanoparticles exposed their prospective effect on the burn wound healing process, Fig. 2 [65].

Polyvinyl Alcohol hydrogels loaded with propolis were prepared by the freeze–thawing process for wound care applications. Antibacterial properties, a barrier to microbial penetration, and releasing propolis of these materials suggest a successful wound healing gel [66]. Table 3 summarizes some recent studies concerning hydrogel propolis composite and its effect and benefit as wound healing material.

**Table 3:** Propolis hydrogel composites and its effect and application for wound healing

Propolis composite	Propolis effect	Applications	References
Bacterial nanocellulose (BNC) hydrogels containing 1.2, 2.4, and 3.6% (w/w) propolis extract and MB photosensitizer	Formulations containing MB and propolis significantly reduced Staphylococcus aureus growth. In the presence of light, BNC/MB hydrogels completely inhibited the microorganism	Application in photodynamic inactivation (PDI), potential materials for the prevention or treatment of *S. aureus* infections in wounds	[67]
Cellulose nanofibers (0.3 g)/poly(vinyl alcohol) (0.3 g), hydrogels functionalized with propolis (0.1–0.2 g)	Hydrogels exhibited a potential antimicrobial effect, the greatest viability, adhesion, and spreading of cells and a significant anti-inflammation potential	Biomedical applications and wound healing	[68]
Chitosan, honey, propolis, and venom hydrogel	Antioxidant activity, which can protect the cells against the damage of oxidation. Inhibiting and clearing the biofilm of the bacterial cocktail	Promising agent against wound infection and enhanced wound healing	[69]
Propolis-loaded PVA hydrogel	Rapid re-epithelialization, significant burn wound contraction	The burn wound healing	[36]
Bandage based on an ethanolic extract of propolis at 15%, 30%, and 60% (w/v); and cellulose acetate hydrogel cross-linked with EDTA	Preventing infections, promoting lesion retraction, and tissue regeneration. Revealing a reduced inflammatory process and stimulating skin regeneration. Accelerating healing process to a full recovery tissue repair after 14 days	Second-degree burns treatment	[70]
Hydrogel films from quaternized chitosan blended with pectin and loaded with propolis	Promote antibacterial activity and cell migration in the scratch assay. Improved the tensile strength and stability of the hydrogel films. Has an antioxidant activity and were non-toxic to L929 cells	Improve wound healing ability, wound dressing materials	[71]

### Natural rubber

The biocompatible Natural Rubber Latex was incorporated with propolis to acquire its antibiotic properties whilst keeping the desirable characteristics of rubber. Natural Rubber membrane loaded with propolis demonstrated antimicrobial activity and optimal controlled release of propolis appropriate for wound healing bandages [72].

Natural rubber membranes associated with aqueous propolis extract are promising as a dressing for wound healing in a rat’s burn model. These curative membranes accelerated the burn healing process and tissue repair [73].

### Cellulose

Cellulose, the most abundant biopolymer on the earth, has emerged as a substitute for fossil fuel-based polymers [74, 75]. Plants usually contain cellulose combined with lignin and other polysaccharides. Cellulose is a linear polysaccharide chain of D-glucopyranose rings connected via β-1,4-glycosidic links [76]. Cellulose fibers have high mechanical strength from the hierarchical structure and hydrogen bridging at the fiber surface [74]. Cellulose is a promising polymer for creating sustainable films and hydrogels, which can be applied in various applications [77, 78]. Bacterial cellulose is a talented safe fabric for wound healing. Various cellulose derivatives, such as carboxymethyl cellulose can be prepared from cellulose, resulting in functionalized and more active polymers [59]. The term “nanocellulose materials” describes the variety of cellulose structures with unique properties of nanocellulose, such as enhanced crystallinity, high surface area, rheological properties, alignment and orientation, biodegradability, biocompatibility, and low toxicity [79]. Biocellulose membranes were prepared and then immersed in propolis to attain bactericide dressings. These membranes were efficient against Staphylococcus species and show, in vivo tests, a superior tissue repair in the healing process. Bacterial cellulose associated with propolis was evaluated as a curative in the diabetic mouse. The produced biocuration was capable of accelerating the wound healing process in a diabetic mice model [80].

Cellulose fibers were blended with poly(vinyl alcohol), a hydrophilic semi-crystalline, water soluble, and non-toxic synthetic polymer. The films loaded with encapsulated vitamin C and propolis may represent a new therapeutic approach to accelerate diabetic wound healing. Films enable the release of vitamin C in a controlled manner. Vitamin C is a well-known natural antioxidant involved in all phases of wound healing, mainly in the collagen formation phase. Propolis can improve some biological mechanisms involved in wound healing (e.g. epithelialization) [81].

### Starch

**Fig. 3:**
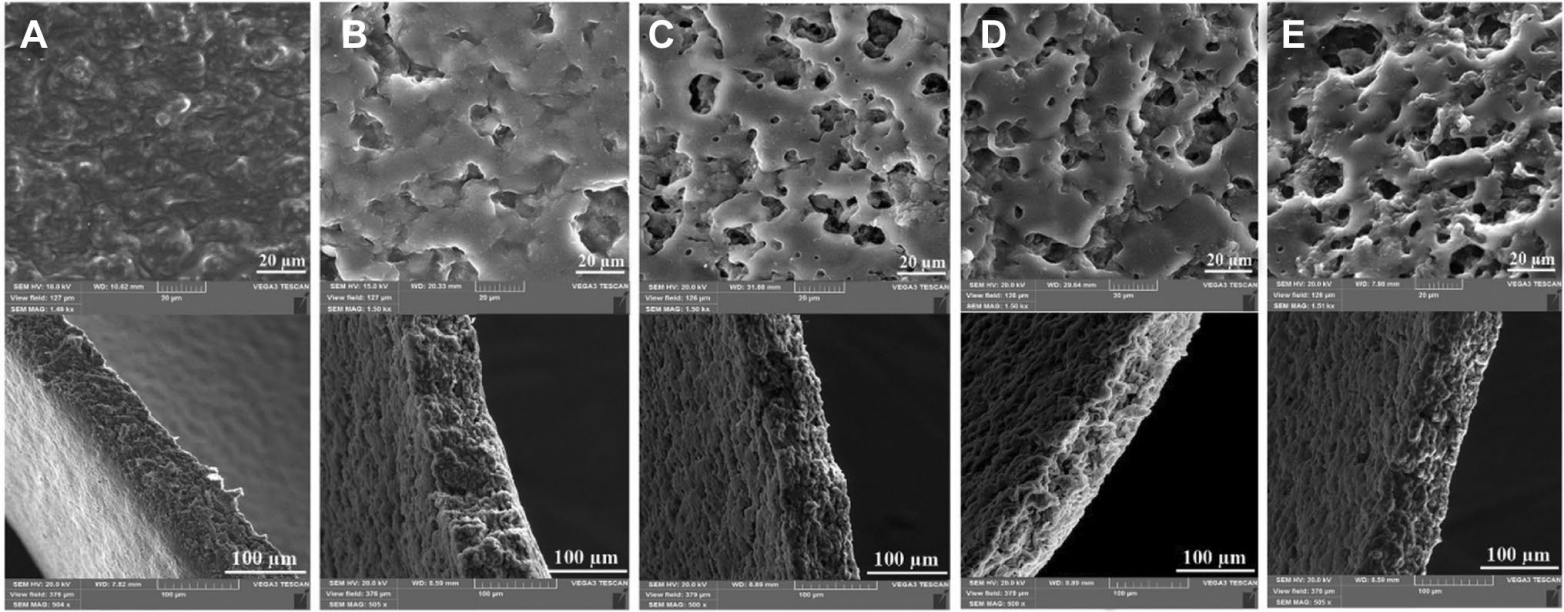
SEM micrographs of the surface (top) and cross-section (down) of cornstarch (**A**), cornstarch/hyaluronic acid (**B**), cornstarch/hyaluronic acid with various ethanol extract propolis 0.25% (**C**), 0.5% (**D**) and 1% (**E**) film dressings [27]

Starch typically consists of two polysaccharides, amylose (a linear chain with few branches), and amylopectin (a highly branched chain). Each starch component has a different effect on the starch’s properties. For example, most amylose chains are shorter than those in amylopectin [82]. Biodegradable, edible films based on starch and propolis have been developed for packaging application technology; propolis nanoparticles may act as a natural bio plasticizer [83–86]. The starch-modified chemistry and many reactive sites can hold biologically active compounds. The solvent-casting method prepared propolis extract blends with starch and hyaluronic acid, as shown in Fig. 3. Cornstarch/hyaluronic film dressings displayed a rough surface. In addition, increased propolis extract concentration increased films’ surface roughness. The results showed that the incorporation of hyaluronic and propolis caused an insignificant effect on the films’ porosity, but considerable differences were seen in the roughness of the film’s surface. According to the cross-sectional SEM micrographs in Fig. 1, the film containing 0.5% propolis showed higher antibacterial activity and no cytotoxicity. It is suggested as a wound dressing material that could successfully accelerate the wound healing process [27]. Active blends from carrageenan and starch-containing natural antioxidants were prepared. New functional films with antioxidant properties are made with yerba mate and Cuban red propolis extracts. The design of polysaccharides blend for the release of the bioactive compound is a promising method to avoid the spoilage of food products [87].

### Synthetic polymers

High porous polyurethane (PU) foams were prepared and coated with propolis to prepare coated wound dressing materials. These composites significantly enhanced in vitro cellular compatibility and in vivo wound healing, directly related to coated propolis concentration. Therefore, the propolis-coated polyurethane wound dressing can be suitable for more pre-clinical investigations [88].

In a different study, biocompatible propolis-loaded polyurethane (propolis/PU) nanofibers were prepared using propolis/PU blend solution electrospinning. The incorporation of propolis into PU fibers improves cell compatibility and antibacterial activity. Therefore, as-synthesized nanocomposite fibrous mat has great potential in wound dressing and skin tissue engineering [48].

Polyvinyl alcohol/propolis scaffolds showed adequate fiber morphology and did not present cytotoxicity to fibroblasts in vitro. The high wound closure rate suggests that PVA scaffold be applied in tissue regeneration [89].

## Conclusion

Propolis has become one of the most attractive natural materials for developing advanced bioactive wound dressings due to its unique properties such as antibacterial, antifungal, antiviral, anti-inflammatory, anticancer, and antitumoral. Propolis could imitate the native skin extracellular matrix structure, recapitulate the wound healing process, and provide biomaterial tunability. Natural polymers, such as cellulose, chitosan, and starch, can provide controllable and effective approaches for propolis. Moreover, natural polymers have high biocompatibility and easily stimulate cell growth and regulation. However, synthetic polymers provide mechanically stable and humid environments to support wound healing and skin regeneration. Film dressings of cornstarch/hyaluronic acid containing EEP were prepared as wound dressings by solvent-casting technique. The films containing 0.5% propolis extract showed higher antibacterial activity and no cytotoxicity and could successfully accelerate wound healing. Cornstarch containing propolis can be pointedly applied as wound dressing material. Electrospun scaffolds can meet most of the essential requirements for accelerated wound healing, including minimizing infections. It remains difficult to deploy these materials on a large-scale-industrial basis, partly due to their low production rates. A fine-tuned control of biodegradation rates of scaffold properties is needed to match the native wound healing process. However, electrospinning-based approaches have a bright future in wound healing and wound care product development, despite some perceived drawbacks.

## Data Availability

All data generated or analyzed during this study are included in this published article.
